# Motor Power Signal Analysis for End-Point Detection of Chemical Mechanical Planarization

**DOI:** 10.3390/mi8060177

**Published:** 2017-06-05

**Authors:** Hongkai Li, Xinchun Lu, Jianbin Luo

**Affiliations:** Department of Mechanical Engineering, Tsinghua University, Beijing 100084, China; xclu@tsinghua.edu.cn (X.L.); luojb@mail.tsinghua.edu.cn (J.L.)

**Keywords:** chemical mechanical planarization, motor power, end-point detection, smooth

## Abstract

In the integrated circuit (IC) manufacturing, in-situ end-point detection (EPD) is an important issue in the chemical mechanical planarization (CMP) process. In the paper, we chose the motor power signal of the polishing platen as the monitoring object. We then used the moving average method, which was appropriate for in-situ calculation process and made it easy to code for software development, to smooth the signal curve, and then studied the signal variation during the actual CMP process. The results demonstrated that the motor power signal contained the end-point feature of the metal layer removal, and the processed signal curve facilitated the feature extraction and it was relatively steady before and after the layer transition stage. In addition, the motor power signal variation of the polishing head was explored and further analysis of time delay was performed.

## 1. Introduction

With the increase in the number of metal layers and wafer size in the integrated circuit (IC) manufacturing, the need for global planarity across the wafer has become increasingly crucial [1]. Chemical mechanical planarization (CMP) [2,3,4] is presently the most widely used planarization technique. It combines mechanical and chemical removal mechanisms in a synergistic effect [3], so that it can offer excellent local as well as global planarity of the wafer surface.

The present technology of IC manufacturing uses copper (Cu) as the interconnect metal because of its lower resistivity [5]. For Cu CMP process, the end-point is defined by the full removal of the extra metal (including the barrier layer) without over-polishing the Cu line inside the vias or contacts, which might cause dishing and erosion [1]. Without assistance of end-point detection (EPD) technology, problems of under-polishing and over-polishing could occur easily. Both conditions require wafers to be reprocessed or discarded, thus reducing the total throughput [5]. Therefore, accurate determination of when a CMP process reaches the end-point has been one of the most important research issues [6].

EPD technology is a big challenge of the CMP process [7], because in-situ EPD is difficult to perform under the actual process conditions [1]. At present, various EPD methods [5,8,9,10,11,12,13,14] have been proposed, such as optical reflectance, frictional force, pad temperature, Cu-ion concentration, and eddy current. In the self-developed CMP system, the eddy current method [13] has been used to develop independently an in-situ measurement module for detecting the Cu layer thickness variation [12]. However, when the thickness of the Cu layer is too small or the local Cu layer has been removed, the sensor is unable to function well. Therefore, it is necessary to establish another method to further improve the existing measurement module for detecting the ideal end-point of the Cu CMP process and overcome the drawbacks of the eddy current method.

In this study, we attempted to use the motor power signal of the polishing platen for detecting the end-point of the metal layer removal during the CMP process. Much work on signal processing and analysis has been done. We expected that the processed signal curve could provide information on layer transition across the entire wafer surface and aid in determining the ideal end-point of the CMP process with a good planarization result.

## 2. Frictional Method

Using motor power signal to achieve in situ EPD is based on the frictional method. Specifically, the hardness of the dielectric layer differs from that of the metal layer, and so do the friction coefficients of these two layers engaged with the pad. When the upper metal layer is removed and the pad starts to polish the underlying dielectric layer, the electric motor has to change the current, voltage, or power to drive the polishing head or the polishing platen with a constant velocity. Therefore, monitoring the change in such variables for EPD is a feasible approach.

Based on the frictional method, the motor current technique [9] has been applied to detect the end-point of metal CMP processes [1]. This technique is commonly employed for tungsten process, and typically requires a physical clamp or a hall-effect probe to detect the motor current change from the polishing head or polishing platen [14]. The system relies on the hardware filters and sophisticated mathematical functions to calculate the end-point accurately. Once the end-point is detected, the polishing process is stopped [15]. In addition, the motor current variation is influenced by not only the polished material but also the pad conditioning or other factors. Therefore, much work should be done to achieve an effective EPD.

In this study, we chose the motor power signal of the polishing platen as the experimental object, instead of motor current. Unlike other proposed methods, we aimed at reducing the complication in the system development and ensured that the data processing method was more efficient under the practical CMP conditions. Finally, we could extract the end-point feature from the smoothed signal curve with ease.

## 3. Experiment and Data Acquisition

### 3.1. Process and Results

**Figure 1:**
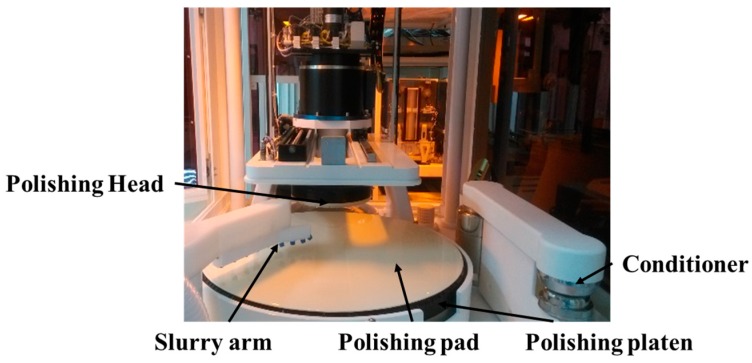
Chemical mechanical planarization (CMP) system used for experiment.

The experiments were performed on the self-developed CMP system, as shown in Figure 1. The pad and slurries used for polishing were commercially available, as shown in Table 1. The experimental samples were 12-inch through-silicon-via (TSV) wafers. The samples were metallized with approximately 4200 nm Cu on top of 350 nm Ti used as a barrier layer. The thickness of tetraethyl orthosilicate (TEOS) used as the dielectric layer was about 2000 nm.

In the CMP process, the change of some process parameters could have effect on the polishing result, as well as the motor power signal, and it was of importance to keep a good flatness of wafer surface. Therefore, the CMP process had been optimized for achieving better polishing results before the actual experiments. All wafers were polished first for the removal of much of the Cu overburden and then for the experiments. In the experiments, the slurry flow rate was 300 mL/min; the rotation speed of the polishing head was 87 rpm; the rotation speed of the polishing platen was 93 rpm; and the polishing pressures of zones 1–4 were 3 psi, with high pressures of zone 5 and the retaining ring. In the paper, zone 1 is the central zone of the polishing head. In the polishing process, the pad was conditioned in situ.

**Figure 2:**
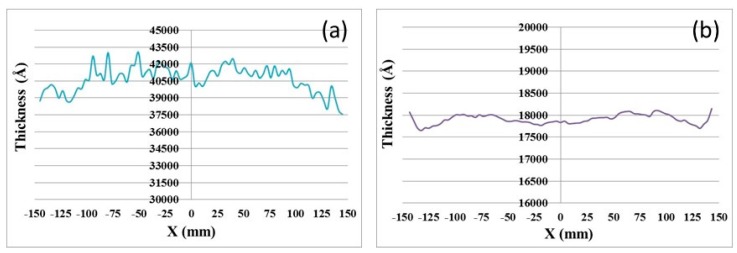
(**a**) Cu thickness profile before CMP process; (**b**) tetraethyl orthosilicate (TEOS) thickness profile after CMP process.

Before the experiment, a four-point probe meter was used to measure the thickness of the Cu layer. After the experiment, a Filmetrics F50 film measurement system was used to measure the thickness of TEOS layer. In this study, the *x*-direction diameter (vertical to the notch) of wafer was chosen to represent the surface layer thickness. Figure 2 shows the measurement results of a wafer before and after the CMP process, and the nonuniformity of the remained dielectric layer was 0.61%, whereas the initial nonuniformity of the Cu layer was 2.85%. In this study, the maximum nonuniformity of the dielectric layer of the processed wafer was less than 1.9%.

**Figure 3:**
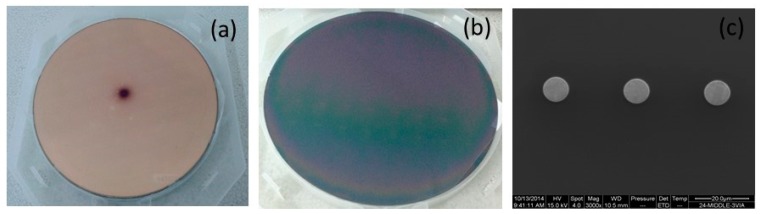
(**a**) Unpolished wafer surface; (**b**) polished wafer surface; (**c**) through-silicon-via (TSV) Cu stud after CMP process.

In this study, a scanning electronic microscopy (SEM, FEI Quanta 200F, Holland, The Netherlands) was used to characterize the Cu surface topography after the experiment. As shown in Figure 3, the surface of the Cu stud obviously showed its color and the barrier layer (Ti) was removed completely. Furthermore, the dishing and erosion on the Cu lines measured by a surface profile topography were much less.

### 3.2. Data Acquisition

In order to acquire the motor power signal without adding new hardware, serial communication was employed. The host (Industrial Personal Computer, IPC) was connected to the motor drive of the polishing platen through RS-232. The link between the host and the drive permitted both sides to communicate using ASCII-coded messages. In this study, the command of the actual motor power was PMECH, and the serial port was opened in the read-write mode. The configuration of the serial port is presented in Table 2. When the program was running, an independent thread was created for long-lasting serial communications with the motor drive. Once valid data are available, the control system read the data and extracted useful information immediately.

**Figure 4:**
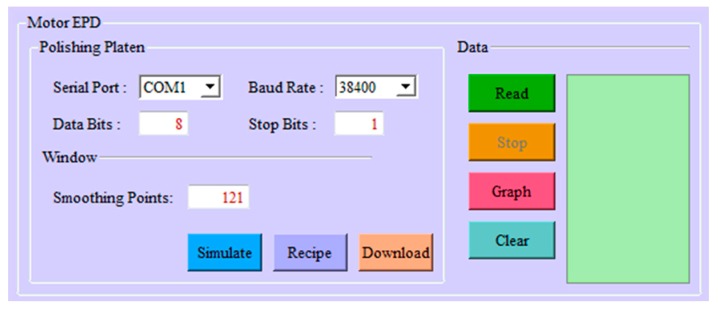
The graphic user interface (GUI) of data acquisition system.

Qt [16] is a leading cross-platform application and graphic user interface (GUI) development framework. It is widely used for developing software applications. Its powerful full-framework capabilities allow for the creation of native applications with high performance. The QtSerialPort module is an add-on module for the Qt5 library. Use of the QtSerialPort module significantly reduces the time for implementing applications that require access to a serial interface. Thus, in the software development process, C++ was chosen as the programming language in combination with the Qt framework to create the GUI shown in Figure 4 and communication module.

## 4. Data Processing

### 4.1. Original Signal

**Figure 5:**
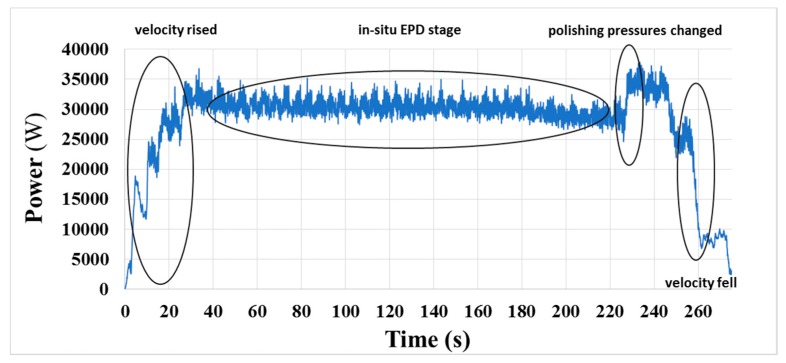
Original motor power signal of the polishing platen drive during a CMP process.

In the CMP process, the acquired original signal generally contained much unwanted noise and fluctuates greatly, which definitely limited its application. If the original motor power signal had slight noise and was consistent from one process to the next, the system could seek an appropriate end-point feature easily and extract it. However, the polishing process comprised the rotation of the polishing platen, rotation and oscillation of the polishing head, and in-situ conditioning, all of which could contribute considerable fluctuations to the original signal, as shown in Figure 5. Therefore, the original signal could not be used directly in the program for in-situ end-point calculation.

The original signal shown in Figure 5 is obtained from the polishing platen drive during an entire CMP process, and the process contains several successive stages. In the program, the main polishing stage is separated from the other stages and set as the in-situ EPD stage, in which the polishing pressure and the rotation speed are constant, so that the influence from the signal variation that is caused by changes in the process parameters of the other stages can be eliminated effectively. Then, we need to process the signal curve and expose the possible end-point feature in the original signal. Some processing methods [6,7,15] reported in the previous works are complicated. In this study, the data processing method should be efficient under practical CMP conditions, and easy for coding.

### 4.2. Data Processing

**Figure 6:**
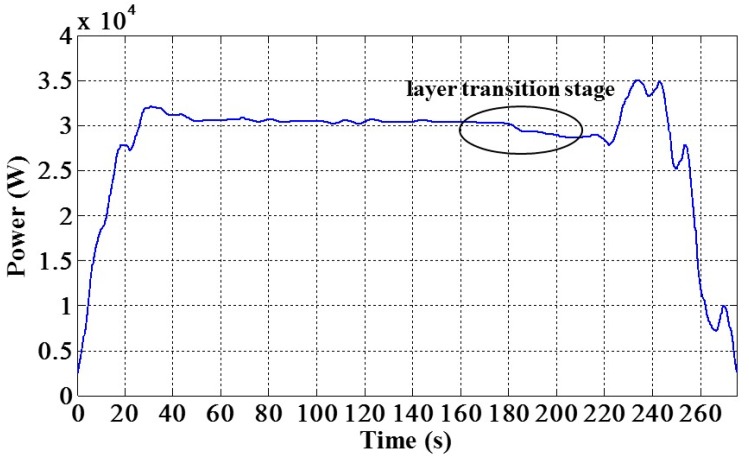
Sixth-level approximation of the motor power signal by wavelet analysis.

In the paper, we first check whether the motor power signal contains the end-point feature. Thus, wavelet analysis [7] is used for analyzing the original signal. In this study, wavelet decomposition was performed off-line, and a db4 wavelet was used. The result was gotten from the sixth-level decomposition. In the experiment, the in-situ EPD stage started at about 20 s and ended at about 220 s. At the beginning of this stage, the polishing pressure was adjusted gradually to the set value, so that a signal rise appeared at that moment. As shown in Figure 6, the circled region revealed the layer transition stage in the CMP process. Therefore, it demonstrates that the motor power signal provides the potential for detecting the end-point of CMP process. Then, we should seek a data processing way which is appropriate for in-situ calculation process.

Compared with other data processing methods, the moving average method, which was dynamic, was used. In this method, a moving window is moved to add new data points along with the data sampling process and the window span is kept constant by removing the old points in the window. The original data are smoothed by replacing each data point with the average of the neighboring data points defined within the window. A detailed description of this strategy is shown as

(1) $$y(i)=\frac{\left[x(i+N)+x(i+N-1)+...+x(i-N)\right]}{2\times N+1}$$

where *y*(*i*) is the smoothed value of the *i*th data point, *N* is the number of neighboring data points on either side of *x*(*i*), 2*N* + 1 is the span of the moving window, and *x*(*i*) is the original value of the *i*th data point.

**Figure 7:**
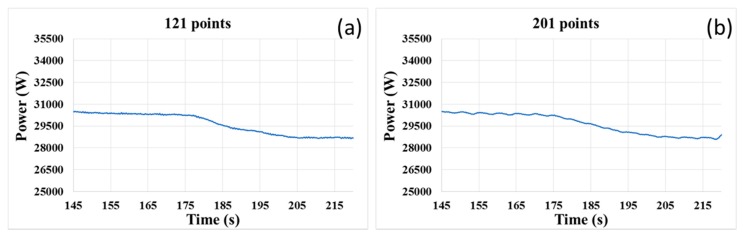
Smoothing results with different span of the moving window: (**a**) 121 points; (**b**) 201 points.

**Figure 8:**
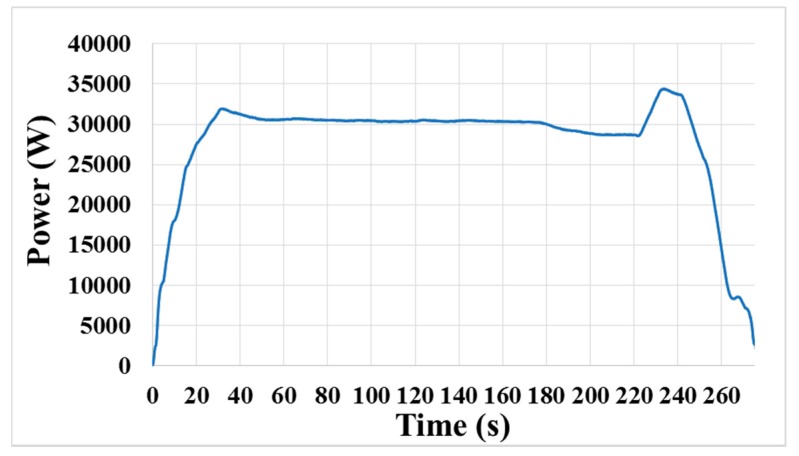
Smoothed motor power signal curve of the polishing platen during a CMP process.

The window span is determined by the user. Generally, the greater the number of points in the moving window is, the smoother the curve is. Thus, it is necessary to broaden the span to achieve a better smoothing result. However, this principle may be not always true within a certain range, according to the obtained results. In Figure 7b, the span of the moving window is 201 points, which is more than that of Figure 7a. It is clearly observed that some small fluctuations exist in the smoothed curve shown in Figure 7b, which makes the smoothing result in this case worse than that in Figure 7a. In addition, too many data points in the moving window can result in a big time delay, and more points would cause an increase in the delay time. Furthermore, the end-point feature will be weakened, if the span of the moving window is too great. Therefore, the span of the moving window should be a modest value. In this study, 121 points within the moving window were enough at such sampling rate. As shown in Figure 8, the smoothing signal curve is obtained by using a 121-point moving average method, and all noise components of the original signal (shown in Figure 5) are removed.

## 5. Analysis and Discussion

### 5.1. Signal Analysis for EPD

In this study, we mainly investigated the signal variation during the layer transition process from the barrier layer to the dielectric layer. During the experiment, the metal layer including the little remained Cu layer and barrier layer (Ti) was removed first, after which the underlying dielectric layer (TEOS) began to be polished. Because the friction coefficients of two layers (Ti and TEOS) engaged with the pad were different, the motor drive had to change the power to drive the polishing platen with a constant velocity. Figure 7a shows the variance of the smoothed power signal before and after the layer transition during the CMP process. It could be clearly observed that the signal decreased as the exposed TEOS layer increased in area and it reached to a new stable value when the Ti layer was cleared completely. Furthermore, the smoothed signal was relatively steady before and after the transition stage, which made it easier and more reliable to detect the layer transition.

As seen in Figure 7a, the motor power decreases substantially from approximately 30,300 W to 28,670 W (i.e., a difference of 1630 W) during the transition region. It is also noted that the signal shows a significant decrease in a relatively long period of time, about 30 s, which is consistent with the removal rate of Ti. Thus, this observation clearly indicates the feasibility of using the smoothed motor power signal curve of the polishing platen to detect the transition from the barrier layer to the subsequent dielectric layer, or the end-point.

**Figure 9:**
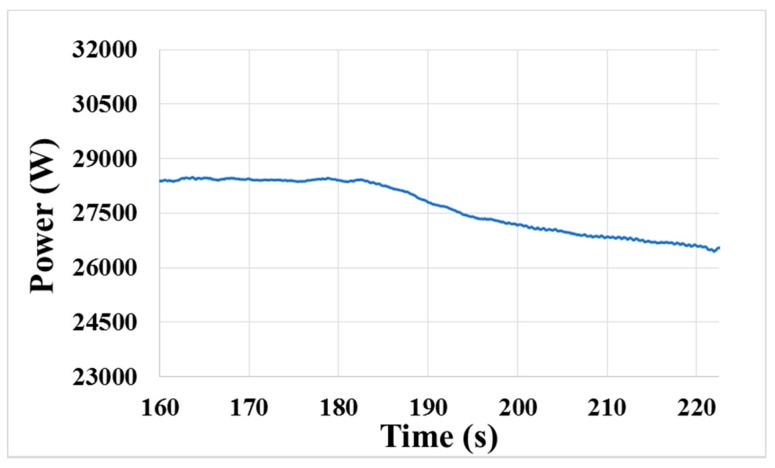
Smoothed signal curve of final experiment during the transition region.

The typical CMP process always suffers from the problem of a decline in the lot-to-lot (even wafer-to-wafer) polishing rate on account of the pad aging and the difference between wafers, which in turn may also affect the stability of EPD system. In this study, we focused on the variation of the motor power during the transition region. In the actual in-situ calculation process, the slope variation of the smoothed signal curve would be used to capture the end-point feature. Therefore, the influence of the pad and wafers could be reduced. After several days of consecutive CMP processes, in the final experiment, a big difference was also observed in the smoothed signal curve before and after the layer transition region, as shown in Figure 9. The transition time was about 37 s, and the power difference could reach up to 1890 W.

**Figure 10:**
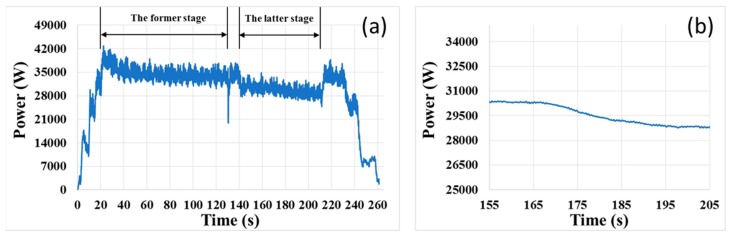
(**a**) Original motor power signal of polishing platen; (**b**) smoothed signal curve of (a) during the transition region.

In addition, we did an experiment, in which the in-situ EPD stage was mainly divided into two stages. The duration of former stage is 110 s, and the rotation speeds of the polishing head and platen were 97 rpm and 103 rpm respectively, which were greater than those of the normal CMP condition; the duration of latter stage is 70 s, and the process parameters were same as the normal CMP condition; and there was a buffer stage with 10 s between the two stages, in which the polishing pressure decreased and then the rotation speeds were back to 87 rpm and 93 rpm. The original signal in the experiment is shown in Figure 10a, and the smoothed signal curve during the transition region is shown in Figure 10b. As seen in Figure 10a, the motor power signal was sensitive to the change in the rotation speed, and the motor power signal decreased to the normal level, when the process was into the latter stage. Furthermore, the end-point feature appeared earlier in the experiment, because the material removal rate in the former stage increased.

### 5.2. Motor Power Signal of the Polishing Head

**Figure 11:**
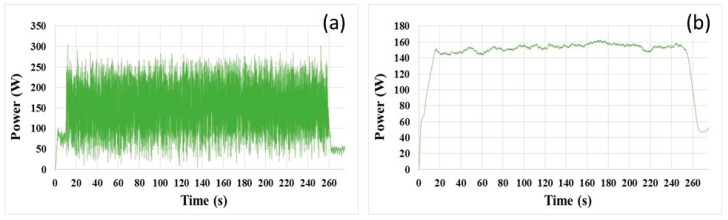
(**a**) Original motor power signal of polishing head; (**b**) smoothed signal curve of (a).

In this study, the motor power signal of the polishing head was not used, because the original power signal of the polishing head contained more noise with large amplitude, as shown in Figure 11a. After the smoothing process, the smoothed curve shown in Figure 11b was still not as stable as that of the polishing platen, when the span of the moving window was 121 points (same as the case of the polishing platen). What was worse, no obvious end-point feature could be extracted.

In the CMP process, the polishing head must stabilize the pressures of all zones in real time, and the inertia of the polishing head is much smaller than that of the polishing platen. Therefore, we think the fluctuation of the polishing pressure has a greater influence on the motor power signal of the polishing head. In future work, we would continue to pay attention on the motor power signal of the polishing head, and study the signal variation in the actual CMP process.

### 5.3. Time Delay

Adopting the existing moving average method would result in a time delay, because it needs the next points for recalculating the present data value. In this study, the span of moving window mainly determined the delay time (denoted as *T*), and the delay time could be estimated by using the sampling rate (denoted as *R*) and the number (denoted as *N*, which was defined in Section 4.2) of data points on one side in the moving window. Specifically, the calculation equation was $T=\frac{N}{R}$. Under the present conditions that the signal sampling rate was 12.15 Hz, the delay time was less than 5 s. At a removal rate of 229 nm/min (when the down force was 3 psi), the over-removed TEOS thickness was estimated to be less than 20 nm, which satisfied the process requirement. In this section, we tried some ways to reduce the time delay. More work on data processing should be done in the further study.

#### 5.3.1. Sampling Rate

**Figure 12:**
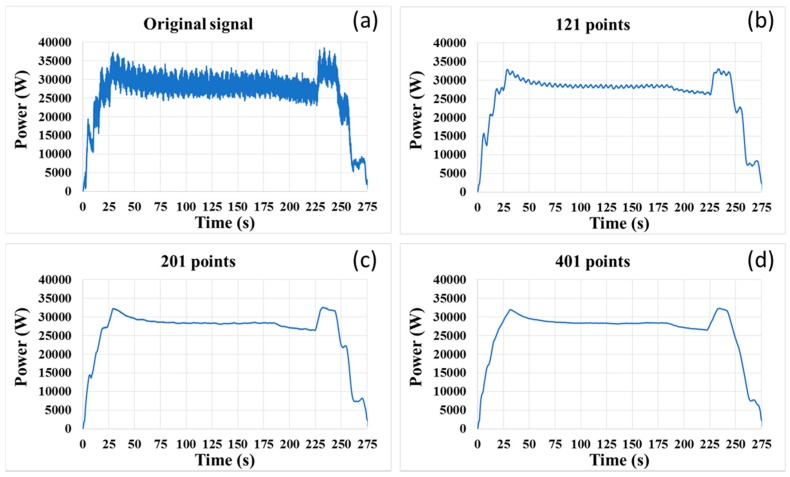
(**a**) Original signal; (**b**) smoothed signal curve with 121-points moving window; (**c**) smoothed signal curve with 201-points moving window; (**d**) smoothed signal curve with 401-points moving window.

On condition that the span of moving window is constant, an increase in the sampling rate will reduce the delay time. In this study, we improved the sampling rate by 3.3 times to 40 samples per second, and the same moving average method was used to smooth the signal curve. However, the smoothing result was worse than what was expected. In practice, more points within the span of the moving window were used for improving the smoothing result. As a result, the delay time did not decrease, even though the sampling rate was improved. As shown in Figure 12, the span of the moving window should be 401 points, if we want to obtain a usable signal curve. In that case, the delay time is 5 s.

#### 5.3.2. Average Method

**Figure 13:**
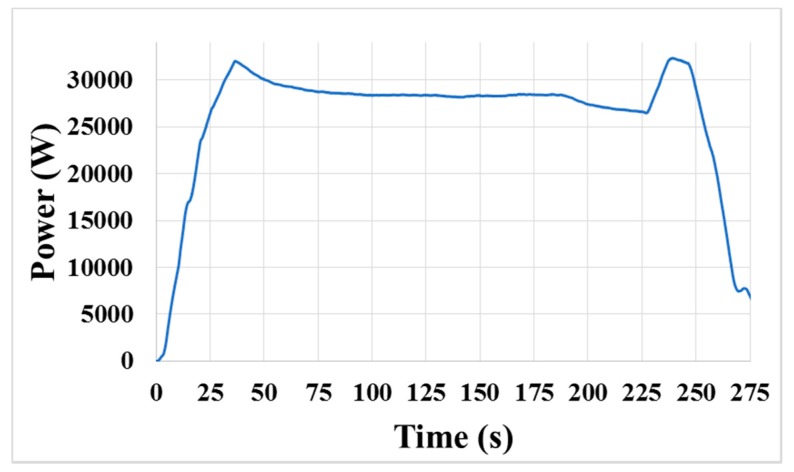
Smoothed signal curve obtained by the revised average method.

The previous average method is to replace each data with the average of the neighboring data points defined within the window. In this section, the average method is revised, and only the previous points are used to calculate the present data point. After recalculating the signal data, it was clearly observed that the transition region began at approximately 189 s, as shown in Figure 13, whereas it started at 183 s in Figure 9. Thus, the beginning time of layer transition is postponed for 6 s by using the revised way. Based on the study above, we would attempt to improve the algorithm of end-point determination to guarantee a good process result.

## 6. Conclusions

In the paper, we studied the variation of the motor power signal of the polishing platen during the CMP process, and specifically described how to acquire, process, and analyze the signal data. Based on the results, it could be clearly observed that the smoothed signal curve decreased as the exposed dielectric layer increased in area and it reached to a new stable value when the metal layer was cleared completely. Furthermore, the smoothed signal curve was relatively steady before and after the transition stage. Therefore, it was demonstrated that the motor power signal of the polishing platen provides the potential for detecting the layer transition from the barrier layer to the dielectric layer, or the end-point in the CMP process, and the moving average method with 121-points moving window was effective and made it easy to extract the end-point feature. In this study, adopting the moving average method could result in a small time delay. In future, more work on the algorithm of end-point determination should be done.

## Figures and Tables

**Table 1:** Specifications of the pad and slurry.

Item	Type
Pad	Politex
Slurry	Anji-Z4U (with 0.15% H_2_O_2_)

**Table 2:** Configuration of the serial communication.

Parameter	Value
BaudRate	38,400
DataBits	8
Parity	No Parity
StopBits	One Stop
FlowControl	No Flow Control
